# General Screening Rules and Segmented Optimization Strategy for Efficient Thermoelectric Devices Validated by Mg_3_(Sb,Bi)_2_/Bi_0.5_Sb_1.5_Te_3_‐GeTe Module

**DOI:** 10.1002/advs.202502832

**Published:** 2025-05-05

**Authors:** Kai‐Yu Yang, Xiaoyuan Li, Yuanxin Jiang, Liangliang Wang, Kai Guo, Lei Miao, Junliang Chen, Jiye Zhang, Lin Li, Yusong Du, Guang‐Hui Rao, Jun Luo, Jing‐Tai Zhao

**Affiliations:** ^1^ School of Materials Science and Engineering Guilin University of Electronic Technology Guilin 541004 China; ^2^ School of Physics and Materials Science Guangzhou University Guangzhou 510006 China; ^3^ Key Lab of Si‐based Information Materials & Devices and Integrated Circuits Design Department of Education of Guangdong Province Guangzhou 510006 China; ^4^ Guangxi Novel Battery Materials Research Center of Engineering Technology State Key Laboratory of Featured Metal Materials and Life‐cycle Safety for Composite Structures School of Physical Science and Technology Guangxi University Nanning 530004 China; ^5^ Guangxi Key Laboratory of Information Materials Guilin University of Electronic Technology Guilin 541004 China; ^6^ School of Materials Science and Engineering Shanghai University Shanghai 200444 China; ^7^ Interdisciplinary Materials Research Center School of Materials Science and Engineering Tongji University Shanghai 201804 China

**Keywords:** compatibility factor, matching relation, relative current density, segmented thermoelectric device, thermoelectric conversion efficiency

## Abstract

Segmentation is a widely adopted strategy to enhance the efficiency of medium‐ and high‐temperature thermoelectric devices by capitalizing on the distinct properties of materials across various temperature ranges. Establishing a highly compatible matching relationship is crucial for maximizing conversion efficiency. In this study, the concepts of materials’ compatibility factor and relative current density into the screening process are emphasized for universal adaptability. The effectiveness and practicality of the theoretical model for optimal segmented combinations are validated through COMSOL finite element simulations and experimental results. A segmented thermoelectric power generation device is constructed that integrates the environmentally friendly n‐type Mg_3_(Sb,Bi)_2_ with the optimal segmented pairing of Bi_0.5_Sb_1.5_Te_3_‐GeTe. Notably, at a temperature difference of Δ*T* = 440 K, this device achieves a maximum conversion efficiency of 10.4% and a peak output power of 0.41 W. These findings provide a solid theoretical foundation for the development of efficient combinations of thermoelectric materials.

## Graphical abstract

By leveraging the materials' compatibility, optimized matching relationships are established to enhance the performance of segmented thermoelectric devices. To validate this approach, a segmented thermoelectric generator is fabricated incorporating n‐type Mg_3_(Sb,Bi)_2_ and p‐type Bi_0.5_Sb_1.5_Te_3_‐GeTe. Remarkably, under Δ*T* = 440 K, the device achieves a maximum energy conversion efficiency of 10.4% and a peak power output of 0.41 W.

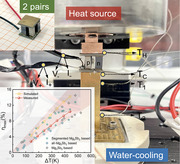

## 1 Introduction

**Figure 1:**
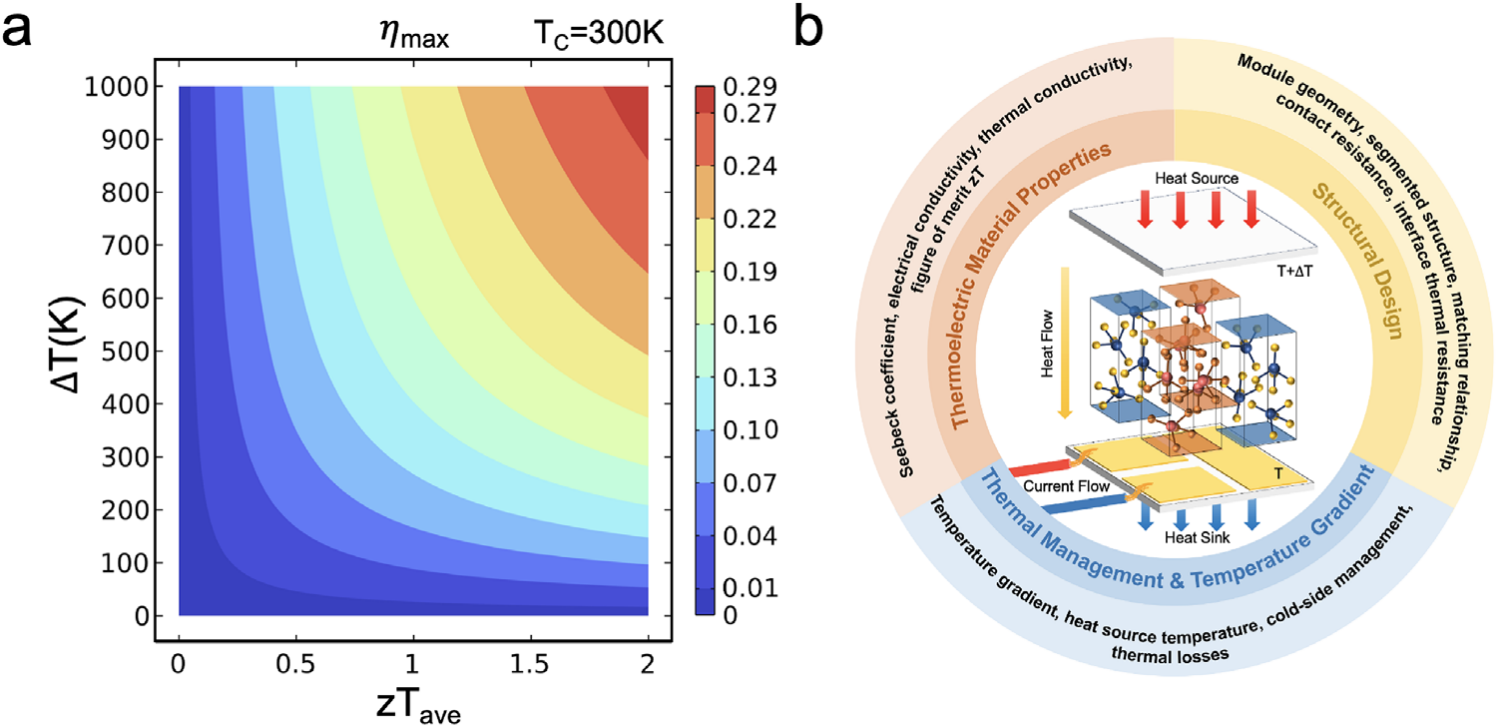
a) The conversion efficiency *η*_max_ for a thermoelectric power‐generation device versus temperature difference and *zT*_ave_. b) The complex factors influencing the real conversion efficiency of a thermoelectric power‐generation device.

With the increasing prominence of renewable energy sources and the emphasis on energy utilization efficiency, the potential applications based on thermoelectric power generation in the realms of waste heat recovery and environmental monitoring have come to the forefront.^[^ ^1^, ^2^, ^3^, ^4^, ^5^, ^6^, ^7^, ^8^ ^]^ The power‐generation capacity depends predominantly on the materials’ thermoelectric figure of merit $\mathrm{zT}=\frac{\alpha^{2}\sigma T}{\kappa }$, where *z*, *α*, *σ*, *T* and *κ* are the thermoelectric quality factor, Seebeck coefficient, electrical conductivity, absolute temperature and thermal conductivity, respectively.^[^ ^9^ ^]^ Similar to other heat engines, their efficiency is constrained by the Carnot cycle. The maximum conversion efficiency *η*_max_ is calculated as $\eta_{\max}=\frac{T_{H}-T_{C}}{T_{H}}\;\left[\frac{\sqrt{1+zT_{\mathrm{ave}}}-1}{\sqrt{1+zT_{\mathrm{ave}}}+T_{C}/T_{H}}\right]$, where *zT*_ave_ denotes the average thermoelectric figure of merit, enabling estimation of thermoelectric performance across the temperature spectrum. *T*_H_ and *T*_C_ are the temperatures of the hot side and the cold side, respectively.^[^ ^10^ ^]^ Thus, higher *zT*_ave_ indicates high conversion efficiency with fixed *T*_H_ and *T*_C_. Additionally, for a constant *zT*_ave_, greater temperature differential between the hot and cold sides, ∆*T*, leads to higher conversion efficiency as well (**Figure** **1**a).

In recent decades, significant advancements in thermoelectric materials have greatly contributed to the development of thermoelectric power generation devices.^[^ ^11^, ^12^, ^13^ ^]^ Many traditional thermoelectric materials, such as Bi_2_Te_3_,^[^ ^14^, ^15^, ^16^ ^]^ PbTe,^[^ ^17^, ^18^ ^]^ and SiGe,^[^ ^19^, ^20^, ^21^ ^]^ have surpassed the peak *zT* of 1. In addition, new high‐performance thermoelectric materials, such as Zintl phases,^[^ ^22^, ^23^, ^24^, ^25^, ^26^, ^27^, ^28^, ^29^ ^]^ Skutterudite,^[^ ^30^, ^31^ ^]^ Half‐Heusler,^[^ ^32^, ^33^, ^34^ ^]^ and Cu_2_S,^[^ ^35^, ^36^, ^37^, ^38^ ^]^ are continually under investigation. Among these, the n‐type Zintl phase compound Mg_3_(Sb,Bi)_2_ has emerged as a promising candidate with outstanding thermoelectric properties. Its application potential spans the low‐ and medium‐temperature range and varies depending on the contents of Bi.^[^ ^39^, ^40^, ^41^, ^42^, ^43^, ^44^, ^45^, ^46^ ^]^ Furthermore, it boasts advantages such as cost‐effectiveness, a wide range of composition options, exceptional mechanical durability, and eco‐friendliness, positioning it as a preferred material for thermoelectric devices.

For near‐room‐temperature thermoelectric devices, researchers often combine thermoelectric materials with high *zT* values and optimize the geometric configuration of thermoelectric modules to enhance the device's output performance.^[^ ^47^ ^]^ However, for medium‐ and high‐temperature thermoelectric devices operating in environments with significant temperature differences, a single material cannot guarantee high‐performance output over a wide temperature range. Therefore, segmented thermoelectric devices are frequently applied using thermoelectric materials with high *zT*_ave_ in different temperature regions. This approach aims to maximize the thermoelectric conversion efficiency of the device by utilizing materials with specific temperature‐dependent characteristics (Figure 1b). So far, huge advancements have been achieved in the research of segmented thermoelectric power generation devices. Based on the reasonable structural design of the numerical analysis model, the extremely low thermal and electrical loss, Zhang et al. successfully prepared a segmented module composed of Bi_2_Te_3_‐based alloy and CoSb_3_‐based filled skutterudite. When working at a temperature difference of 814 K, the efficiency was as high as 12%.^[^ ^48^ ^]^ Li et al. demonstrated a segmented bismuth telluride/half‐Heusler device structure, which achieved a high conversion efficiency of 12% at a temperature difference of 584 K.^[^ ^49^ ^]^ Sun et al. prepared a (Bi,Sb)_2_Te_3_/Mg_3_(Bi,Sb)_2_ thermoelectric segmented module by rational design of materials to devices. When the temperature difference was 380 K, the power generation efficiency reached 10.5%.^[^ ^50^ ^]^

While much attention was paid to the relationship between the thermoelectric efficiency of segmented devices and temperature‐dependent thermoelectric properties, the compatibility matching between various materials was rarely investigated although it was very critical. Therefore, it is still necessary to evaluate the compatibility of the most advanced thermoelectric materials. As early as 2003, Snyder and Ursell proposed that, in addition to the quality factor *z*, controlling the compatibility factor *s*, $s=\frac{\sqrt{1+\mathrm{zT}}-1}{\alpha T}$, was also essential for the efficient operation of thermoelectric devices. The compatibility factor *s* aided in the rational selection of materials, device design and engineering of functionally graded materials. They demonstrated that during thermoelectric power generation, only the thermoelectric power generation device composed of segmented thermoelectric materials with the matching compatibility factor could achieve the optimal efficiency due to the limitation imposed by relative current density.^[^ ^51^ ^]^ Snyder further explained the distinctions between compatible and incompatible systems, and provided examples of efficiency reductions resulting from improper segmentation methods. For instance, a segmented combination of p‐type (AgSbTe_2_)_0.15_(GeTe)_0.85_ with its compatible p‐type PbTe segmentation achieved the maximum conversion efficiency of 10.33%. In contrast, using the incompatible SiGe alloy would reduce the efficiency to 9.85%.^[^ ^52^ ^]^ Recently, Lobato et al. conducted related research on the compatibility of thermoelectric materials. They evaluated the efficiency and self‐compatibility of non‐segmented p‐type and n‐type thermoelectric legs through a 1D model, using the most advanced *zT* values to evaluate the efficiency and compatibility of segmented thermoelectric legs composed of two materials.^[^ ^53^ ^]^ This work provides a more comprehensive and detailed strategy for evaluating compatibility. However, the absence of experimental validation underscores the importance of achieving consistency between experimental and predicted outcomes in order to effectively demonstrate the accuracy of this evaluation strategy.

Based on the aforementioned research findings, we innovatively propose a material screening rule based on relative current density and compatibility factor. By accurately screening material combinations with similar compatibility factors, the efficient and cooperative work of segmented design in each temperature range is ensured. In addition, we manufactured thermoelectric power generation devices containing two sets of n‐type Mg_3_(Sb,Bi)_2_ and p‐type Bi_0.5_Sb_1.5_Te_3_‐GeTe thermocouples using the identified best material combination for experimental validation. The experimental results indicated a significant enhancement in the overall thermoelectric conversion efficiency of the segmented power generation device after compatibility and matching optimization. At a temperature difference of 440 K, the maximum conversion efficiency reached 10.4%, and the corresponding peak output power was 0.41 W, which emphasized the effectiveness of the compatibility evaluation process and beneficial for development of efficient thermoelectric devices.

## 2 Results and Discussion

**Figure 2:**
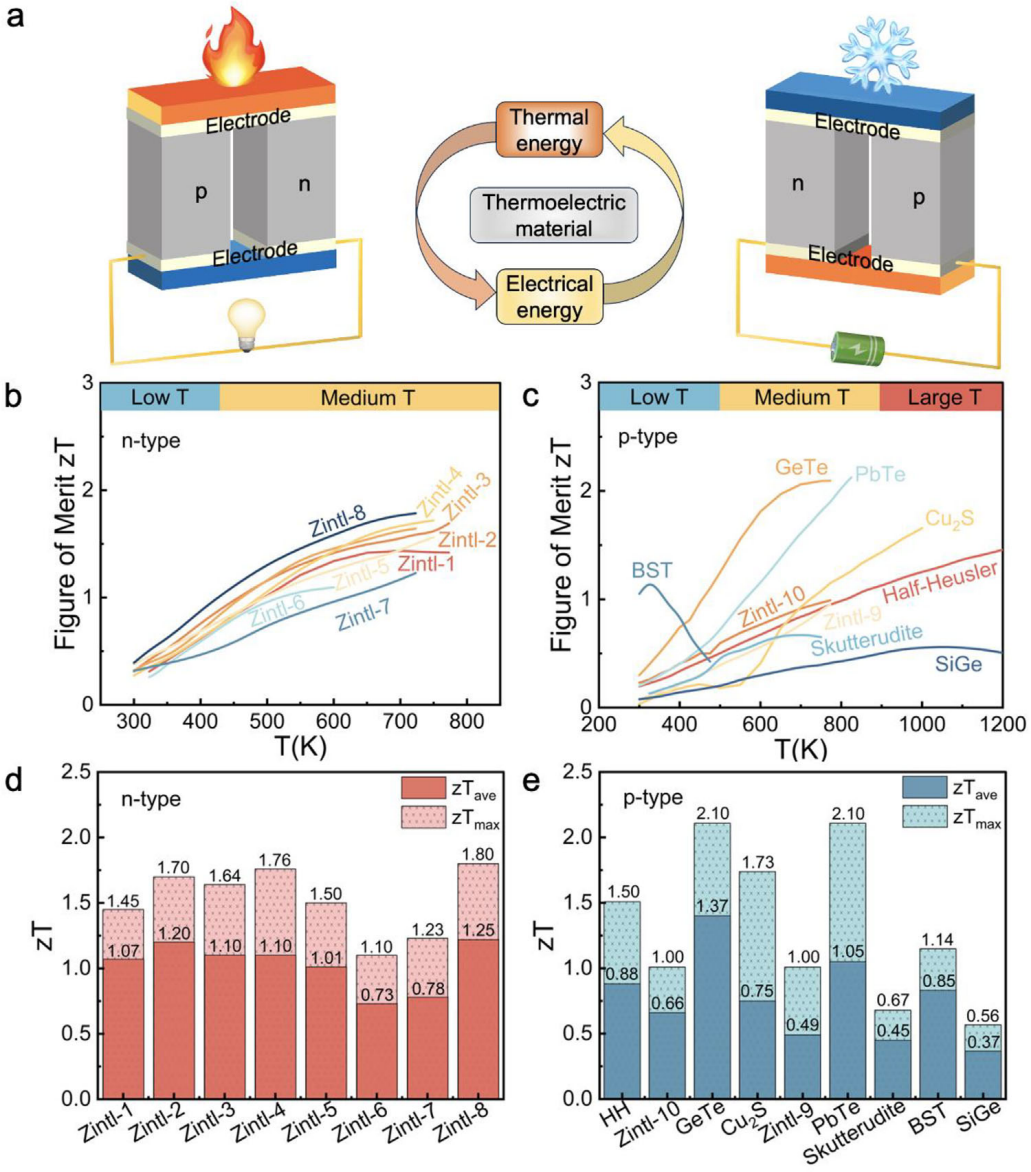
a) The diagrammatic sketch of thermoelectric power generator and thermoelectric cooler. Figure of merit *zT* of all b) n‐type and c) p‐type thermoelectric materials in the material library. Average *zT* (*zT*_ave_) and peak *zT* (*zT*_max_) of these d) n‐type and e) p‐type materials.

Tables S1 and S2 and Figure S1 (Supporting Information) summarize advanced thermoelectric materials and their thermoelectric properties across various temperature regions. The p‐type materials include state‐of‐the‐art Bi_0.5_Sb_1.5_Te_3_ (BST), GeTe,^[^ ^54^ ^]^ and PbTe compounds,^[^ ^18^ ^]^ Zintl phases,^[^ ^25^, ^26^ ^]^ Skutterudite,^[^ ^31^ ^]^ Half‐Heusler (HH) compounds,^[^ ^33^ ^]^ Cu_2_S,^[^ ^38^ ^]^ and SiGe,^[^ ^21^ ^]^ with detailed temperature‐dependent thermoelectric properties shown in **Figure** **2**. The n‐type library features Mg_3_(Sb,Bi)_2_‐based materials,^[^ ^39^, ^40^, ^41^, ^42^, ^43^, ^44^, ^45^, ^46^ ^]^ excelling in middle and low‐temperature ranges, while the p‐type library comprises materials carefully matched to n‐type Mg_3_(Sb,Bi)_2_ for optimal performance. Each material offers distinct advantages in its temperature range, enabling broad applications in thermoelectric power generation and cooling (Figure 2a). In particular, Bi_0.5_Sb_1.5_Te_3_‐based alloys excel near room temperature with the highest *zT* values, dominating thermoelectric cooler applications. GeTe exhibits superior thermoelectric properties compared to other materials in the middle and high‐temperature region, especially in its high‐temperature cubic phase, which matches well with n‐type Mg_3_(Sb,Bi)_2_‐based in terms of thermoelectric performance.^[^ ^40^ ^]^ These two materials not only possess excellent thermoelectric properties within the medium temperature range, but also have comparable thermal expansion coefficients, making them suitable as complementary materials for thermoelectric devices (Table S3, Supporting Information). p‐type Mg_3_Sb_2_‐based materials also perform well near room temperature, enabling homogeneous devices with n‐type Mg_3_(Sb,Bi)_2_ for enhanced stability. Medium‐high temperature materials like Skutterudite, Half‐Heusler, and SiGe show promise for waste heat recovery, power generation, and aerospace applications.

To comprehensively evaluate the thermoelectric properties of materials and guide the segmentation of p‐type materials and n‐p matching, we compiled data on *zT*, *zT*_ave_, and *zT*_max_ (Figure 2b–e). The n‐type Zintl‐8 shows outstanding performance, making it the optimal choice for the n‐leg in thermoelectric devices. Among p‐type materials, Bi_0.5_Sb_1.5_Te_3_ exhibits the highest *zT* in the low‐temperature range (300–400 K), peaking at 1.14 at 325 K, highlighting its potential for low‐temperature applications. GeTe outperforms other materials in the 400–773 K range, achieving a peak *zT* of 2.10 and *zT*_ave_ of 1.37, making it ideal for medium‐high temperature applications. Cu_2_S achieves a remarkable *zT*_max_ of 1.73 at 1000 K, higher than Half‐Heusler at 1200 K. However, Cu_2_S experiences a significant *zT* decline between 300–650 K, leading to a lower *zT*_ave_ (0.75) compared to Half‐Heusler (0.88). This observation underscores the importance of considering the thermoelectric properties of materials across different temperature ranges when selecting and optimizing thermoelectric materials.

As aforementioned, the efficiency of a thermoelectric power generation device is largely determined by two key factors: the temperature gradient across the device and the thermoelectric performance of the materials used. The temperature difference between the two sides of the device establishes the upper limit of the efficiency as expressed by Carnot efficiency *η*_c_, $\eta_{c}=\frac{T_{H}-T_{C}}{T_{H}}$.^[^ ^52^ ^]^ Furthermore, the actual efficiency of the device in comparison to the ideal Carnot efficiency can be assessed through the reduced efficiency *η*_r_, $\eta =\eta_{c}\eta_{r}$, which is influenced by the material's thermoelectric properties, such as the relative current density *u* (the ratio of the electric current density to heat flux by the thermal conductor: $u=\frac{J}{\kappa \nabla T}$) and the quality factor *z* ($z=\frac{\alpha^{2}\sigma }{\kappa }$). The value of the relative current density at the peak of the reduced efficiency during the power generation process is defined as the compatibility factor of the thermoelectric material, denoted as $s=\frac{\sqrt{1+\mathrm{zT}}-1}{\alpha T}$. Both *s* and *zT* are dependent on the material's temperature variation and are derived from its thermoelectric properties represented by *α*, *σ*, and *κ*. As a result, *s* remains constant regardless of device geometry, current, or heat flux.^[^ ^46^ ^]^ To accurately compute thermoelectric efficiency, thermoelectric compatibility is crucial and must be taken into account. The optimal thermoelectric efficiency is typically achieved when *u = s*. Moreover, in segmented thermoelectric power generation devices, due to the limitations of *u*, the best efficiency can only be reached if the segmented thermoelectric materials possess the same compatibility factor *s*. If the compatibility factor varies by a factor of two or more, one cannot apply the same relative current density to both materials simultaneously, rendering segmentation ineffective. Additionally, different characteristics between segments of thermoelectric materials can significantly impact conversion efficiency and output power. Hence, in order to realize high efficiency in n‐type Mg_3_(Sb,Bi)_2_‐based device, the goal is to identify p‐type thermoelectric materials with similar compatibility factor.

**Figure 3:**
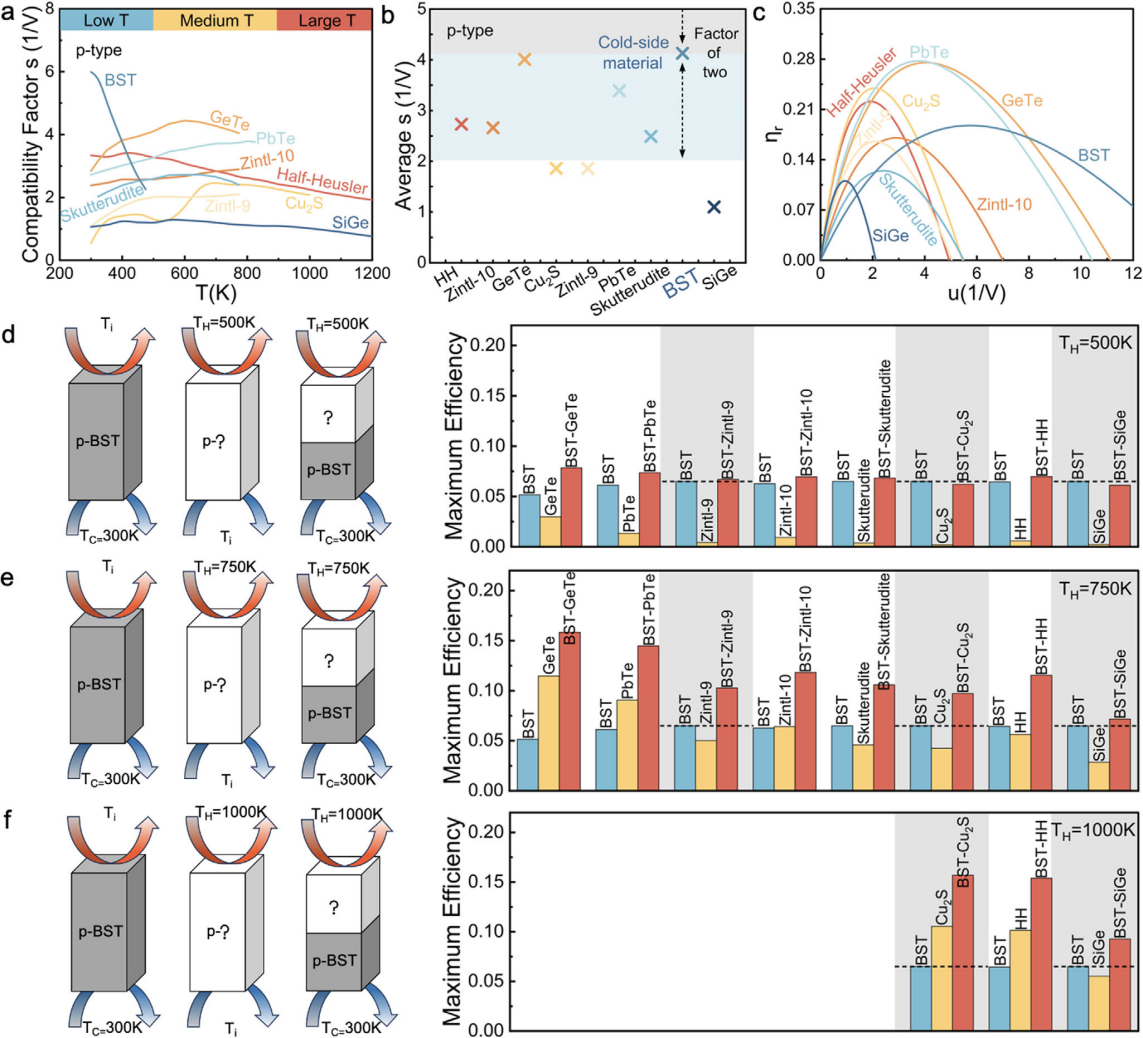
Comparison of a) compatibility factor, b) average compatibility factor over the reported temperature range, c) reduced efficiency for all materials in the p‐type material library. Schematic diagram and maximum efficiency comparison of p‐type cold side material BST single leg, p‐type hot side material single leg, and p‐type segmented single leg at three different hot side temperatures d) 500 K, e) 750 K and f) 1000 K. It is worth noting that the reduced efficiency in (c) is compared at the temperature corresponding to the peak *zT* of each material. The area where the data are not displayed in (f) is due to the temperature exceeding the temperature range reported by the hot side material, and the gray shadow area is the material combination corresponding to the average compatibility factor in (b) exceeding the range of twice.

The compatibility factor (*s*) of all thermoelectric materials in the p‐type material library is presented in **Figure** **3**a. Among them, BST stands out due to its significantly different overall compatibility factor, which offers a more intuitive assessment of both beneficial and ineffective segmentation. While BST demonstrates excellent performance at low temperatures, its compatibility factor is highly temperature‐dependent, potentially complicating effective segmentation. However, as shown in Figure 3b, the average compatibility factors of all p‐type materials across the entire temperature range indicate that when BST is selected as the cold side material, it still holds promise for effective segmentation with most other materials. We highlight the blue shaded area to emphasize that the compatibility factor can help identify a suitable hot side material for beneficial segmentation within a two‐fold range. The average compatibility factor simplifies the process of screening for compatible materials. Nevertheless, the selection of hot‐side materials should not rely solely on comparisons of average compatibility factors. The variation in a material's compatibility factor across the actual temperature range is also critical, especially in applications characterized by significant temperature gradients. For example, while the average compatibility factors of Zintl‐9 and Cu_2_S are slightly greater than twice that of BST, their compatibility factors, as shown in Figure 3a, are similar to those of the cold side material at higher temperatures. This means that a non‐segmented single leg in the mid‐ to high‐temperature regions can still enhance the overall matching effect and facilitate beneficial segmentation. In such scenarios, evaluating the performance of the material post‐segmentation becomes particularly crucial.

At a constant temperature, the reduced efficiency (*η*_r_) fluctuates with relative current density (*u*), as shown in Figure 3c, following a pattern akin to the relationship between total efficiency or power output and current. When *u* approaches the optimal value, *η*_r_ reaches its peak, marking the point where *u* value equals *s*. This indicates that the thermoelectric leg operates under the optimal conditions, allowing for the attainment of maximum thermoelectric conversion efficiency. As *u* deviates from this optimal value, whether increasing or decreasing, *η*_r_ starts to decrease rapidly. These underscores that the thermoelectric leg can only attain its highest efficiency within a specific range of *u*. If *u* exceeds or falls below this range, the performance of the thermoelectric leg will significantly diminish. Figure 3c illustrates that the cold side material BST has the broadest range of relative current density, while the hot side material SiGe has the narrowest. Their optimal *u* values differ significantly, creating a substantial mismatch when combining them. Adjusting *u* for SiGe to its optimal range significantly reduces the efficiency of BST, and vice versa. This makes it challenging to achieve optimal performance for both materials simultaneously, undermining the potential benefits of a segmented design. As a result, the overall efficiency is lower than expected. Therefore, when designing segmented thermoelectric devices, it is crucial to consider the compatibility between the compatibility factor and relative current density. Prioritizing material combinations with similar compatibility factors ensures that each material can operate at its optimal current density, thereby enhancing the overall device efficiency.

Figure 3d–f assesses the impact of combining cold side material BST with various p‐type materials on the maximum efficiency of segmented configurations. Performance is compared to non‐segmented single‐leg designs, and the hot side material is selected based on efficiency evaluation. The calculation results are obtained using the finite element method in COMSOL Multiphysics simulation software at hot side temperatures of 500, 750, and 1000 K, respectively. In the design of segmented single leg, the interface temperature (*T*_i_) plays a crucial role as it determines the temperature range of the hot and cold side materials, which, in turn, affects the comprehensive performance of temperature‐related parameters such as α, σ and κ of each material. Rational selection of the interface temperature *T*_i_ allows each material to operate within its optimal temperature range, optimizing the performance output of the entire single leg. The interface temperature is chosen based on the intersection of the *zT* values (Figure 2c) of the cold and hot side materials; if no intersection is found, the highest temperature of the cold side material is used. The selected interface temperatures are 410, 450, 458, 470, and 475 K.

For the non‐segmented p‐type BST single leg, maximum efficiencies of 5.16%, 6.12%, 6.26%, 6.44%, and 6.51% are achieved at *T*_C_ = 300 K and *T*_H_ = *T*_i_, respectively. From the results in Figure 3d, the efficiency improvement for the segmented single leg is limited, especially for BST‐Zintl‐9, BST‐Cu_2_S, and BST‐SiGe combinations, where the segmented efficiency is slightly lower than that of the unsegmented BST single leg. This is primarily due to excellent thermoelectric properties of BST at lower temperatures (500 K), while the hot‐side material has not fully utilized its advantages in its optimal range. Consequently, at this temperature, the segmented design fails to demonstrate significant advantages. As the hot side temperature increases to 750 K and 1000 K (Figure 3e,f), the relative length of the hot side material increases, allowing it to exhibit its best thermoelectric performance in a higher temperature region. This leads to notable efficiency improvements with the segmented design. Similarly, for BST‐Zintl‐9 and BST‐Cu_2_S legs, the benefits of segmentation are beginning to emerge. However, for the BST‐SiGe legs, the improvement of efficiency is insignificant. This result confirms that the optimal relative current density for SiGe and BST differs significantly, as does the compatibility factor. Thus, there is no suitable current that effectively operates both parts, resulting in poor performance matching at the interface and thus limiting the efficiency improvement of the segmented single leg. In contrast, the BST‐GeTe leg shows significant efficiency improvements, even at *T*_H_ = 500 K, outperforming all other segmented combinations. At *T*_H_ = 750 K, the efficiency reaches 15.84%. This high efficiency can be attributed not only to the excellent thermoelectric properties of GeTe itself but also to its nearly equal average compatibility factor with BST (Figure 3b). The compatibility matching allows the BST‐GeTe combination to easily achieve the best efficiency output, fully leveraging the advantages of the segmented structure.

**Figure 4:**
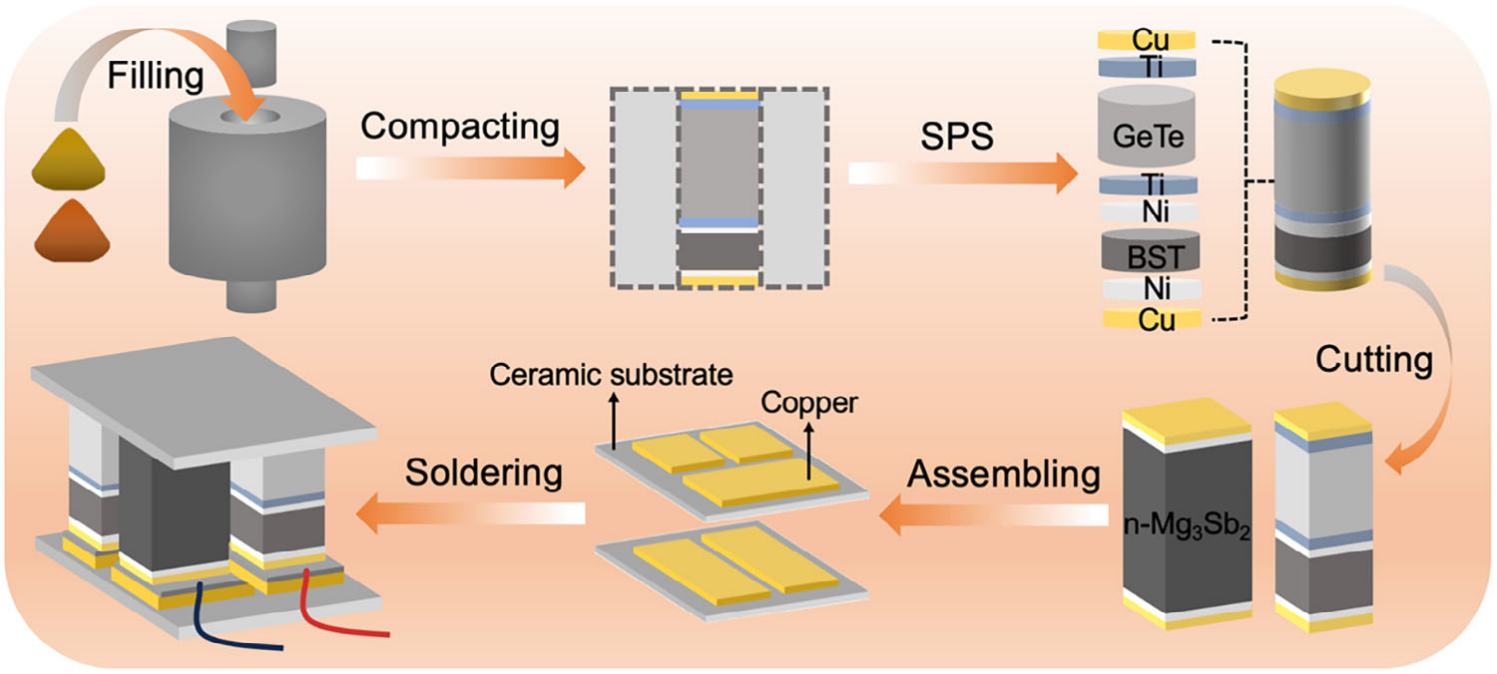
Preparation process of segmented thermoelectric power generation device.

The Zintl‐8, exhibiting the best thermoelectric performance among n‐type materials, is paired with segmented BST‐GeTe optimized for two‐stage temperature intervals, significantly enhancing *zT*_ave_ and expanding the operational range (300–773 K) compared to single‐material systems (Figure S2, Supporting Information). By designing the geometric structure of the device, including the cross‐sectional area ratio (*A*_p_ */A*_n_) and height‐to‐area ratio (*H/A*_pn_), the heat and electrical losses are reduced to further improve the performance (Figures S3 and S4, Supporting Information).^[^ ^55^, ^56^ ^]^ When *A*_p_ */A*_n_ = 0.64 and *H/A*_pn_ = 0.46 mm^−1^, the best conversion efficiency and power density can be achieved. Figure S5 (Supporting Information) shows the segmented unit consistently outperforms non‐segmented and pre‐optimized units, particularly at high temperatures, emphasizing the advantages of segmentation and geometric optimization. Based on this simulation result, the dimensions of (BST)_97_(FeTe_2_)_3_ and (Ge_0.91_Sb_0.09_Te)_0.99_(InSe)_0.01_ legs are adjusted to 3.2 × 3.2 mm^2^ in our segmented thermoelectric power generation device, while the dimension of Mg_3.175_Mn_0.025_Sb_1.5_Bi_0.49_Te_0.01_ is set as 4 × 4 mm^2^. The heights of both thermoelectric legs are determined to be 7 mm. The preparation process of the segmented thermoelectric power generation device is displayed in **Figure** **4**, which includes crucible steps such as material filling, compaction, spark plasma sintering (SPS), cutting, assembly, and welding. Thermoelectric powder, metallized layer powder, and Cu powder were sequentially filled into a graphite mold, with each layer compacted before adding the next. The segmentation ratio of (BST)_97_(FeTe_2_)_3_ and (Ge_0.91_Sb_0.09_Te)_0.99_(InSe)_0.01_ was optimized through finite element simulations (Figures S6 and S7, Supporting Information), and the powder filling adhered strictly to this ratio. After spark plasma sintering, the layered samples were cut into p‐type BST‐GeTe and n‐type Mg_3_(Sb,Bi)_2_ legs. These legs were assembled with ceramic substrates featuring copper electrodes and welded to fabricate the segmented thermoelectric device. Performance was measured by connecting electrical contacts to the first and last legs.

**Figure 5:**
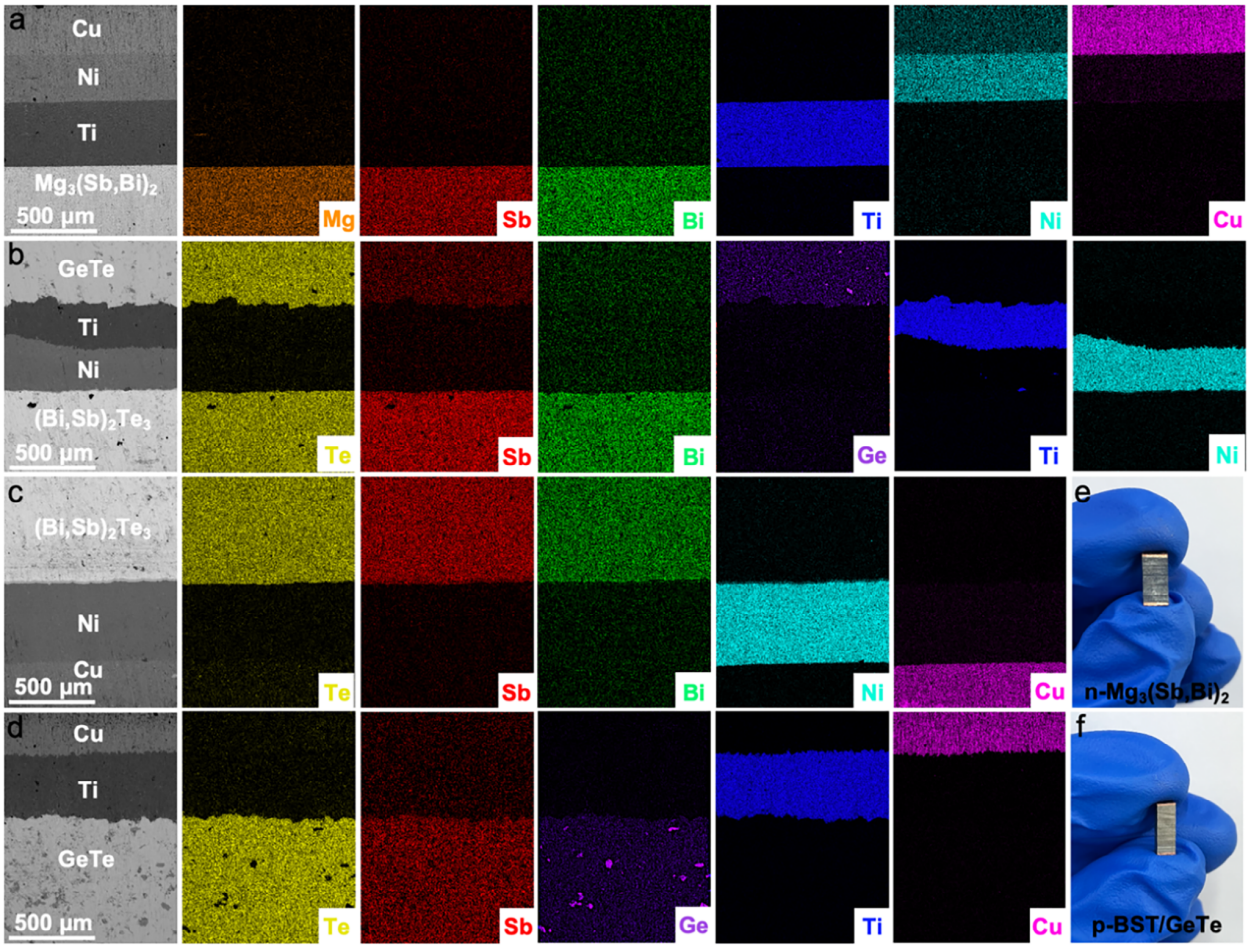
SEM micrograph and EDS mapping of interface layer for a) Mg_3_(Sb,Bi)_2_ leg and b–d) BST‐GeTe leg. The images of e) n‐type Mg_3_(Sb,Bi)_2_ and f) p‐type BST‐GeTe.

**Figure 6:**
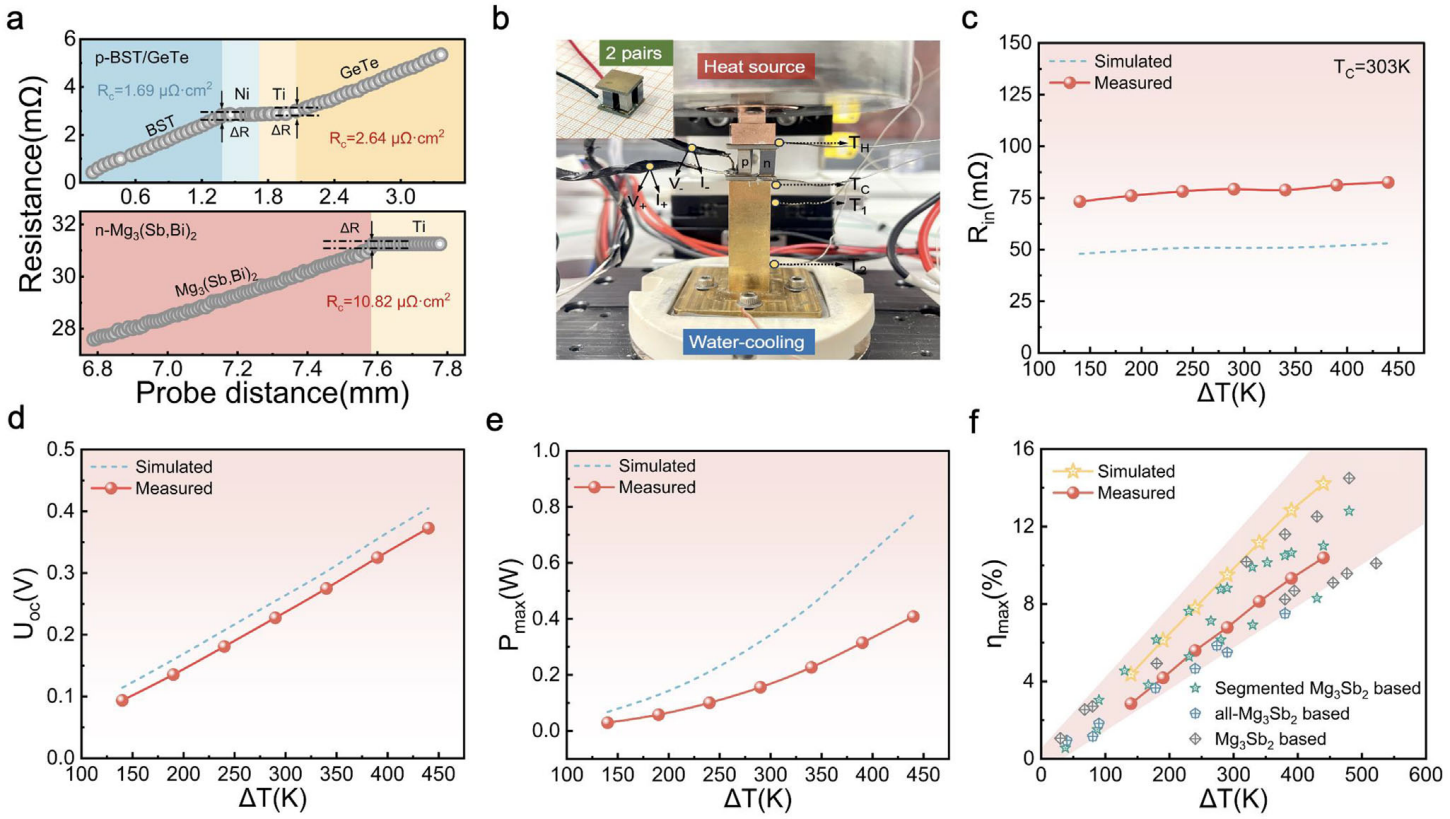
a) The actual interface resistance of n‐type and p‐type thermoelectric legs. b) The energy conversion efficiency test system and the physical diagram of device. c) The total internal resistance (*R*_in_), d) open circuit voltage (*U*_oc_), and e) maximum output power (*P*_max_) of the segmented device under varying temperature differentials. f) maximum conversion efficiency (*η*_max_) of the segmented Mg_3_(Sb,Bi)_2_/BST‐GeTe module measured, in comparison with reported *η*_max_ values for Mg_3_Sb_2_ based modules,^[^ ^55^, ^59^, ^60^ ^]^ all‐Mg_3_Sb_2_ based modules,^[^ ^61^, ^62^ ^]^ and some segmented Mg_3_Sb_2_ based modules.^[^ ^12^, ^40^, ^50^, ^57^ ^]^

The typical barrier layer materials, nickel (Ni) and titanium (Ti), were employed in the thermoelectric legs of the thermoelectric device. The interfaces of the barrier layer were characterized using scanning electron microscopy (SEM) and energy dispersive spectroscopy (EDS), as can be seen in **Figure** **5**. It demonstrates that Ti and Mg_3_(Sb,Bi)_2_ materials exhibit a strong connection, without visible cracks near the interface of the barrier layer.^[^ ^57^ ^]^ Furthermore, the boundary appears clear and smooth, with the thickness of the interface reaction layer less than 1 µm, evidencing high interface stability. Similarly, the lower diffusion rate of Ti effectively inhibits mutual diffusion between the copper electrode and GeTe material.^[^ ^58^ ^]^ No significant element diffusion or reaction was observed at the interfaces as shown in Figure 5b,d. As illustrated in Figure 5b,c, the Ni barrier layer is dense and uniform with a relatively flat interface. Although a thin diffusion layer exists between Bi_0.5_Sb_1.5_Te_3_ and Ni, its thickness remains below 10 µm, which still reflects reliable barrier characteristics. Figure 5e,f shows n‐type Mg_3_(Sb,Bi)_2_ and p‐type BST‐GeTe thermoelectric legs. Contact resistance between metal electrodes and semiconductor materials, primarily exhibiting ohmic behavior, was assessed using a four‐probe method. Voltage fluctuations at the interfaces revealed minimal contact resistance, with interfacial resistivities of 10.82, 2.64, and 1.69 µΩ·cm^2^ for Ti/Mg_3_(Sb,Bi)_2_, Ti/GeTe, and Ni/Bi_0.5_Sb_1.5_Te_3_, respectively (**Figure** **6**a).

The power generation performance of the segmented Mg_3_(Sb,Bi)_2_/Bi_0.5_Sb_1.5_Te_3_‐GeTe thermoelectric device was evaluated. The total internal resistance of the 2‐pair module at room temperature was 68 mΩ, including contributions from electrodes, wires, and welding. Figure 6b illustrates the detailed energy conversion efficiency testing system along with the device itself. The changes of voltage (*U*), output power (*P*), heat flow out of the cold‐side (*Q*_c_), and conversion efficiency (*η*) of 2‐pair module with respect to *I* are shown in Figure S8 (Supporting Information). Key parameters such as *R*_in_, *U*_oc_, *P*_max_, *η*_max_ and *Q*_c_ under varying ∆*T* are illustrated in Figure 6c–f and Figure S9 (Supporting Information). The cold side temperature was maintained at 303 K, while the hot side ranged from 443 to 743 K. Experimental results slightly underestimated simulated predictions due to idealized simulation conditions. As ∆*T* increased from 140 to 440 K, *R*_in_ rose from 73.24 to 82.56 mΩ due to changes in the material's conductivity. At ∆*T* = 440 K, the device achieved optimal values of 0.37 V (*U*_oc_), 0.41 W (*P*_max_), and 10.4% (*η*_max_). The unsegmented BST and GeTe devices were also fabricated and tested, and compared with the segmented device, validating the effectiveness of the segmented evaluation strategy and highlighting the significant advantages of the segmented device over a wide temperature range (Figure S10, Supporting Information).

Furthermore, the maximum conversion efficiency of the prepared Mg_3_(Sb,Bi)_2_/BST‐GeTe module remains at an advanced level when compared to currently reported single‐stage and multi‐stage power generation devices (Figure 6f). This study highlights the significant potential of segmented devices for practical applications and validates the viability of segmented optimization design in assembling cross‐temperature modules.

## 3 Conclusion

To achieve high‐performance thermoelectric devices, segmented combinations of thermoelectric materials are widely utilized. However, rational screening rules and segmentation optimization remain underexplored. In this study, we established the relationship between the compatibility factor and efficiency through COMSOL simulations and experimental validation. Using GeTe as the hot‐side material, which matches well with the compatibility factor of Bi_0.5_Sb_1.5_Te_3_, we achieved a significant efficiency improvement across a wide temperature range. The optimized segmented device reached a maximum efficiency of 10.4% and a peak output power of 0.41 W under a 440 K temperature difference, surpassing single‐material devices. These findings highlight the crucial role of material matching and interface engineering in thermoelectric device design.

## 4 Experimental Section

### Synthesis

*Synthesis of n‐Type Mg_3_(Sb,Bi)_2_‐Based Materials*: Magnesium powders (99.5%, Aladdin), bismuth pellets (99.99%, Aladdin), antimony pellets (99.999%, Aladdin), tellurium pellets (99.999%; Aladdin), and manganese powders (99.99%, Aladdin) were utilized as raw materials, which were weighed in the glove box according to the nominal composition Mg_3.175_Mn_0.025_Sb_1.5_Bi_0.49_Te_0.01_. The mixtures were loaded into a stainless‐steel ball mill tank and milled in an argon (Ar) environment for 10 h. The as‐obtained powders were loaded into a graphite mold, and were subsequently sintered by spark plasma sintering (SPS) under the temperature of 873 K and the pressure of 50 MPa for 10 min.^[^ ^45^ ^]^

*Synthesis of p‐Type Bi_0.5_Sb_1.5_Te_3_‐Based Materials*: Bismuth pellets (99.99%, Aladdin), antimony pellets (99.999%, Aladdin), tellurium pellets (99.999%, Aladdin), and iron powders (99.95%, Aladdin) were accurately weighed according to the nominal composition (BST)_97_(FeTe_2_)_3_ inside a glove box. These materials were then placed into a vacuum‐sealed quartz tube, which was heated to 1173 K at a rate of 2 K min^−1^. After being maintained at this temperature for 24 h, the sample was gradually cooled to 893 K within 2.5 h, remaining at this temperature for an additional 24 h. After cooling to 863 K over 25 h and then to room temperature, the samples were ball‐milled at 800 rpm for 8 min in an Ar atmosphere. The powders were compacted using SPS at 673 K, 50 MPa, for 5 min in a 12.7 mm graphite mold.

*Synthesis of p‐Type GeTe‐Based Materials*: According to the stoichiometric ratio of (Ge_0.91_Sb_0.09_Te)_0.99_(InSe)_0.01_, germanium powders (99.999%, Aladdin), antimony pellets (99.999%, Aladdin), tellurium pellets (99.999%), Aladdin, indium powders (99.99%, Aladdin) and selenium powders (99.999%, Aladdin) were weighed. The raw materials were sealed in a vacuum quartz tube, which was heated at 1223 K for 1 h, annealed for 1 h, and quenched into ice water. The samples were then annealed at 873 K for 72 h. The obtained ingots were ground into powders by high‐energy ball milling for 10 min and hot‐pressed at 823 K for 10 min under a uniaxial pressure of 60 MPa.^[^ ^54^ ^]^

*Fabrication of the Mg_3_(Sb,Bi)_2_/Bi_0.5_Sb_1.5_Te_3_‐GeTe Module*: Using the same sintering conditions as n‐type Mg_3.175_Mn_0.025_Sb_1.5_Bi_0.49_Te_0.01_, n‐type Cu/Ni/Ti/Mg_3.175_Mn_0.025_Sb_1.5_Bi_0.49_Te_0.01_/Ti/Ni/Cu legs were fabricated via one‐step sintering at 923 K, 50 MPa, for 10 min. Cu foil served as the electrode layer, Ni foil as the metallization layer, and Ti foil as the barrier layer, ensuring reliable welding to copper‐coated alumina ceramic substrates. Similarly, p‐type (BST)_97_(FeTe_2_)_3_‐(Ge_0.91_Sb_0.09_Te)_0.99_(InSe)_0.01_ legs were sintered at 723 K, 60 MPa, for 10 min, using high‐purity Cu, Ni, and Ti powders for the layers. The materials were placed into a die in the proper sequence: Cu/Ni/(BST)_97_(FeTe_2_)_3_/Ni/Ti/(Ge_0.91_Sb_0.09_Te)_0.99_(InSe)_0.01_/Ti/Cu. Sintered samples were cut into 4 × 4 × 8 mm^3^ (n‐type) and 3.2 × 3.2 × 8 mm^3^ (p‐type) legs, assembled, and welded with substrates. A thermoelectric power generation device, comprising two thermocouples with dimensions of 12 × 12 × 10 mm^3^, was fabricated. The resistance and contact resistivity values are listed in Table S4 (Supporting Information).

### Characterizations

The electrical conductivity and Seebeck coefficient of bulk materials were measured using a ZEM‐3 instrument (ULVAC, Japan). Thermal conductivity was calculated using *κ* = *dC*_p_ *D*, where *C*_p_ is specific heat capacity, *d* is density (measured via the Archimedes method), and *D* is thermal diffusivity. Microstructures and diffusion layers were analyzed using SEM (Tescan Mira4) with EDS (Xplore 30 EDS). Interface contact resistivity was assessed with a four‐probe system, and power generation performance was tested using Mini‐PEM (Puri Material). Water cooling introduced thermal convection and radiation, potentially causing slight efficiency deviations.

### 3D Finite Element Analysis of Device Performance

The solid heat transfer module and current module in the COMSOL Multiphysics software were utilized to assess the thermoelectric performance of the power generation device. The theoretical simulation, conducted under steady‐state conditions, involved evaluating the output voltage, output power, input heat flow, and conversion efficiency. Thermal convection and thermal radiation were not taken into consideration during the simulation. The device model used in the simulation process is depicted in Figure S11 (Supporting Information), and the parameters for the performance simulation of the 2‐pair module are presented in Table S5 (Supporting Information). Inside the thermoelectric module, the *π*‐type thermoelectric bridge leg was connected using a copper electrode, which was then covered by ceramic substrates. The simulation parameters utilized default values from the COMSOL Multiphysics software package and incorporated material properties characteristic of the thermoelectric material. The visual simulation results of the 2‐pair module are illustrated in Figure S12 (Supporting Information).

## Conflict of Interest

The authors declare no conflict of interest.

## Supporting information



Supporting Information

## Data Availability

The data that support the findings of this study are available from the corresponding author upon reasonable request.
