# Supplementary material for: General Screening Rules and Segmented Optimization Strategy for Efficient Thermoelectric Devices Validated by Mg3(Sb,Bi)2/Bi0.5Sb1.5Te3‐GeTe Module
Source: Adv Sci (Weinh). 2025 May 5;12(27):2502832. doi: 10.1002/advs.202502832 (PMC12279248; doi:10.1002/advs.202502832)
Supplement: Supplementary file 1 — Supporting Information [file ADVS-12-2502832-s001.docx]

**General screening rules and segmented optimization strategy for efficient thermoelectric devices validated by Mg_3_(Sb,Bi)_2_/Bi_0.5_Sb_1.5_Te_3_-GeTe module**

Kai-Yu Yang ^a^, Xiaoyuan Li ^a^, Yuanxin Jiang ^b^, Liangliang Wang ^b^, Kai Guo ^b,c^ *,

Lei Miao ^d^, Junliang Chen ^a,e^, Jiye Zhang ^f^, Lin Li ^a,e^, Yusong Du ^a,e^, Guang-Hui Rao ^a,e^, Jun Luo ^g^ *, Jing-Tai Zhao ^a,e^ *

^a^ School of Materials Science and Engineering, Guilin University of Electronic Technology, Guilin 541004, China

^b^ School of Physics and Materials Science, Guangzhou University, Guangzhou 510006, China

^c^ Key Lab of Si-based Information Materials & Devices and Integrated Circuits Design, Department of Education of Guangdong Province, Guangzhou 510006, China

^d^ Guangxi Novel Battery Materials Research Center of Engineering Technology, State Key Laboratory of Featured Metal Materials and Life-cycle Safety for Composite Structures, School of Physical Science and Technology, Guangxi University, Nanning 530004, China

^e^ Guangxi Key Laboratory of Information Materials, Guilin University of Electronic Technology, Guilin 541004, China

^f^ School of Materials Science and Engineering, Shanghai University, Shanghai 200444, China

^g^ Interdisciplinary Materials Research Center, School of Materials Science and Engineering, Tongji University, Shanghai 201804, China

**Corresponding author*: kai.guo@gzhu.edu.cn (K. Guo); junluo@tongji.edu.cn (J. Luo); jtzhao@guet.edu.cn (J.-T. Zhao)

This supplement contains

Supplementary Tables S1-S5

Supplementary Figure S1-S12

Supplementary References

**Supplemental Tables**

**Table S1.** Advanced n-type thermoelectric materials, showing the material category, composition and temperature range.

| N-type materials | Compositions | T (K) |
| --- | --- | --- |
| Zintl-1: Mg_3_Sb_2_ | Cu_0.03_Mg_3.4_Sb_1.5_Bi_0.49_Te_0.01_ | 323-773 |
| Zintl-2: Mg_3_Sb_2_ | Mg_3.1_Co_0.1_Sb_1.5_Bi_0.49_Te_0.01_ | 300-773 |
| Zintl-3: Mg_3_Sb_2_ | Mg_3.17_In_0.03_Sb_1.5_Bi_0.49_Se_0.01_ | 300-723 |
| Zintl-4: Mg_3_Sb_2_ | Mg_3.2_Y_0.04_La_0.01_Sb_1.5_Bi_0.5_ | 300-750 |
| Zintl-5: Mg_3_Sb_2_ | Mg_3.3_Sb_1.49_Bi_0.49_Te_0.02_ | 300-750 |
| Zintl-6: Mg_3_Sb_2_ | Mg_3.05_La_0.005_Sb_1.5_Bi_0.5_ | 325-600 |
| Zintl-7: Mg_3_Sb_2_ | Mg_3.07_Sb_1.5_Bi_0.48_Se_0.02_ | 300-723 |
| Zintl-8: Mg_3_Sb_2_ | Mg_3.175_Mn_0.025_Sb_1.5_Bi_0.49_Te_0.01_ | 300-723 |

**Table S2.** Advanced p-type thermoelectric materials, showing the material category, composition and temperature range.

| P-type materials | Compositions | T (K) |
| --- | --- | --- |
| Metal tellurides: Bi_0.5_Sb_1.5_Te_3_ | (BST)_97_(FeTe_2_)_3_ | 300-475 |
| Metal tellurides: GeTe | (Ge_0.91_Sb_0.09_Te)_0.99_(InSe)_0.01_ | 300-773 |
| Metal tellurides: PbTe | Pb_0.96_Tl_0.02_Na_0.02_Te | 300-825 |
| Zintl-9: Mg_3_Sb_2_ | Mg_2.095_Yb_0.3_Cd_0.6_Na_0.005_Sb_2_ | 300-773 |
| Zintl-10: CaMg_2_Bi_1.98_ | Ca_0.65_Yb_0.35_Mg_1.9_Zn_0.1_Bi_1.98_ | 300-773 |
| Skutterudite | Dy_0.7_Co_2.5_Fe_1.5_Sb_12_ | 323-723 |
| Sulfide: Cu_2_S | Cu_1.97_S | 300-1000 |
| Half-Heusler (HH) | FeNb_0.88_Hf_0.12_Sb | 300-1200 |
| SiGe | Si_0.8_Ge_0.2_ | 300-1200 |

**Table S3.** Coefficient of thermal expansion of several typical thermoelectric materials.

| **Material** | **Range (K)** | **α_L_ (**$\text{×}$**10^-6^ K^-1^)** |
| --- | --- | --- |
| n-type Mg_3_Sb_2_-based | 300-773 | 22.3^[1]^ |
| p-type cubic GeTe-based | 300-773 | 23.4^[2]^ |
| p-type Bi_2_Te_3_-based | 300 | 19^[3]^ |
| p-type Mg_3_Sb_2_-based | 300-773 | 22.3^[1]^ |
| p-type PbTe-based | 373-773 | 20^[3]^ |
| p-type nanostructured SiGe | 323-1273 | 4.6^[4]^ |
| p-type ZrCoSb | 300-1073 | 7.5^[5]^ |
| p-type Ce_0.9_Fe_3_CoSb_12_ | 300-900 | 12^[3]^ |

**Table S4.** Resistance of each thermoelectric joint and contact resistivity of each interface.

| **Thermoelectric joints** | **Dimension**  **(mm^3^)** | **Resistance**  **(mΩ)** | **Contact resistivity**  **(μΩ·cm^2^)** |
| --- | --- | --- | --- |
| Ti/Mg_3_(Sb,Bi)_2_/Ti | 4 $\text{×}$ 4 $\text{×}$ 8 | 16 | 10.82 |
| Ni/BST/Ni/Ti/GeTe/Ti | 3.2 $\text{×}$ 3.2 $\text{×}$ 8 | 14 | 1.69, 2.64 |

**Table S5.** Parameters for 2-pair module performance simulation.

| **Parameters** | **Value** |
| --- | --- |
| pairs | 2 |
| p-type legs | 3.2 mm $\text{×}$ 3.2 mm |
| n-type legs | 4 mm $\text{×}$ 4 mm |
| H_TE_ | 7 mm |
| AlN L_c_ | 12 mm $\text{×}$ 12 mm |
| AlN H_c_ | 0.5 mm |
| AlN Ceramic | Selected from material library |
| Cu L_e_ | 10 mm $\text{×}$ 4 mm/4 mm $\text{×}$ 6 mm/4 mm $\text{×}$ 11.6 mm |
| Cu H_e_ | 0.3 mm |
| Cu electrodes | Selected from material library |
| T_H_ | 443 K-743 K |
| T_C_ | 303 K |

**Supplemental Figures**


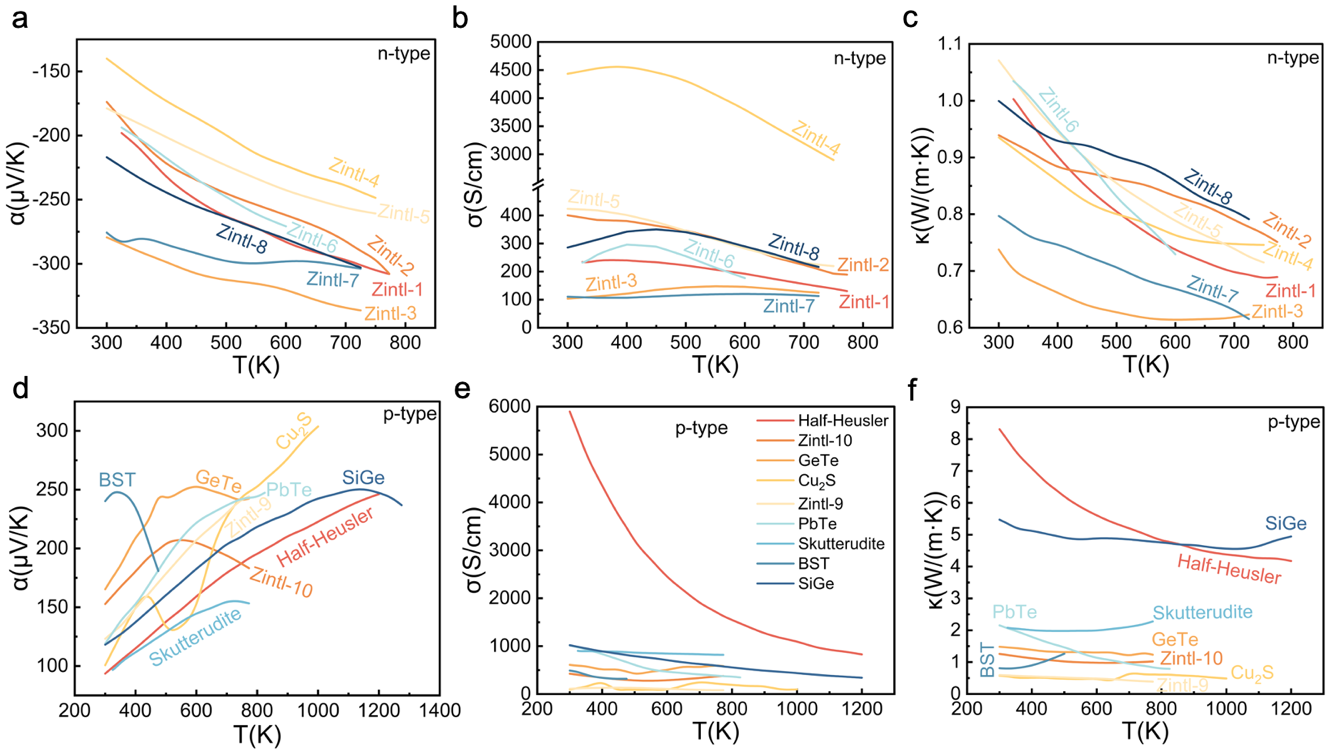


**Fig. S1.** Temperature-dependent thermoelectrical properties of the selected n-type and p-type thermoelectric materials. (a), (d) Seebeck coefficient. (b), (e) Electrical conductivity. (c), (f) Total thermal conductivity.


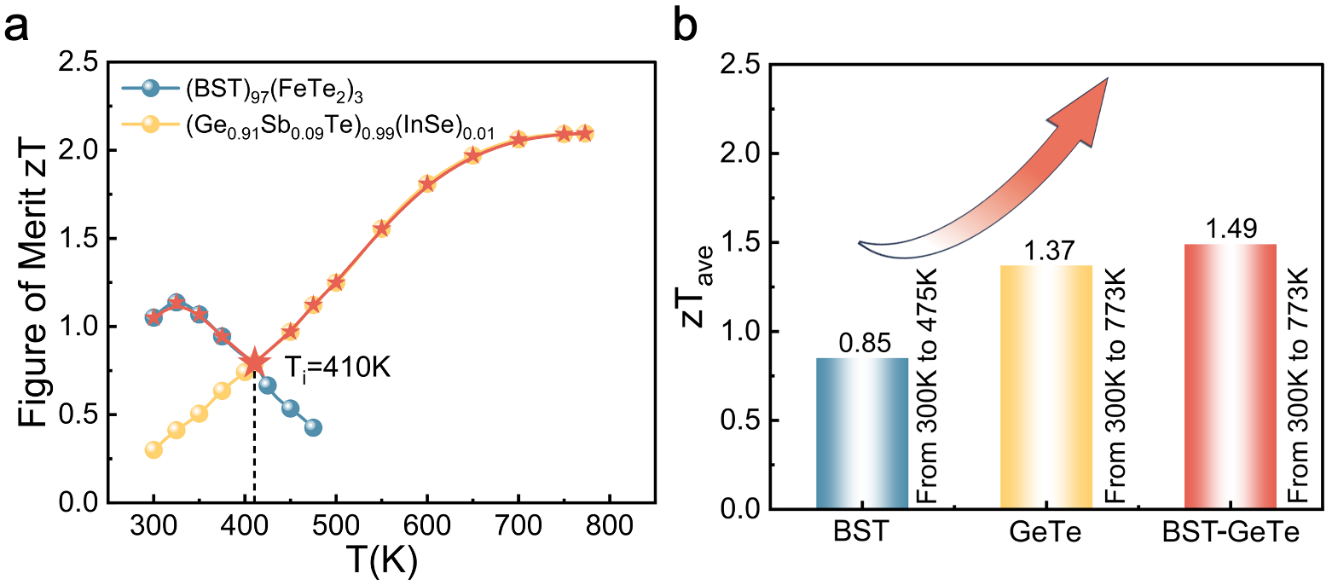


**Fig. S2.** (a) *zT* for p-type BST and GeTe in different temperature ranges. The red lines represent the *zT* of their respective optimal temperature ranges. (b) Average thermoelectric figure of merit *zT*_ave_.


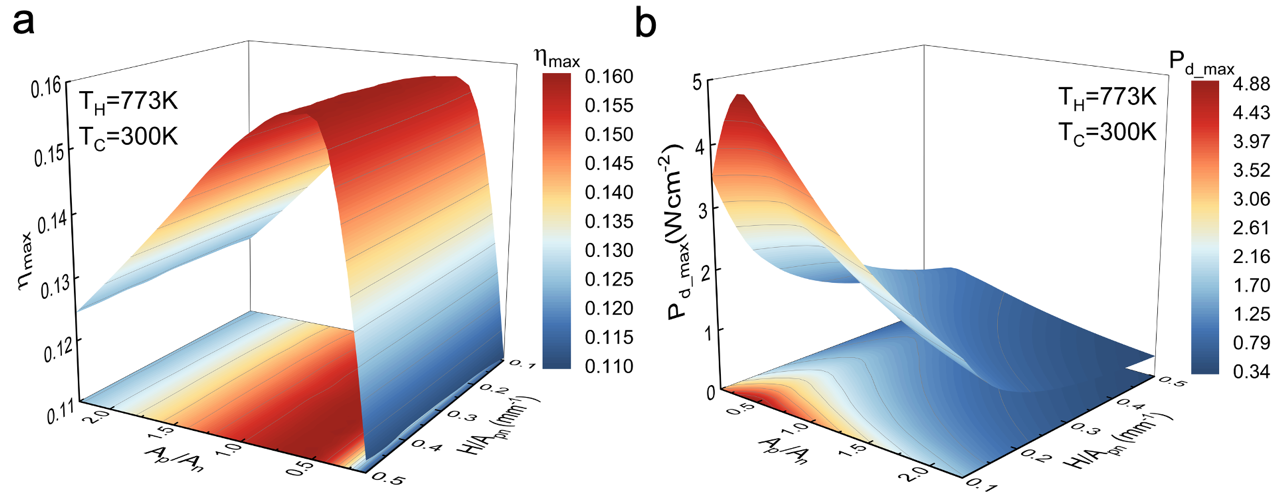


**Fig. S3.** 3D diagram of (a) maximum conversion efficiency $\eta$_max_ and (b) maximum power density *P*_d_max_.


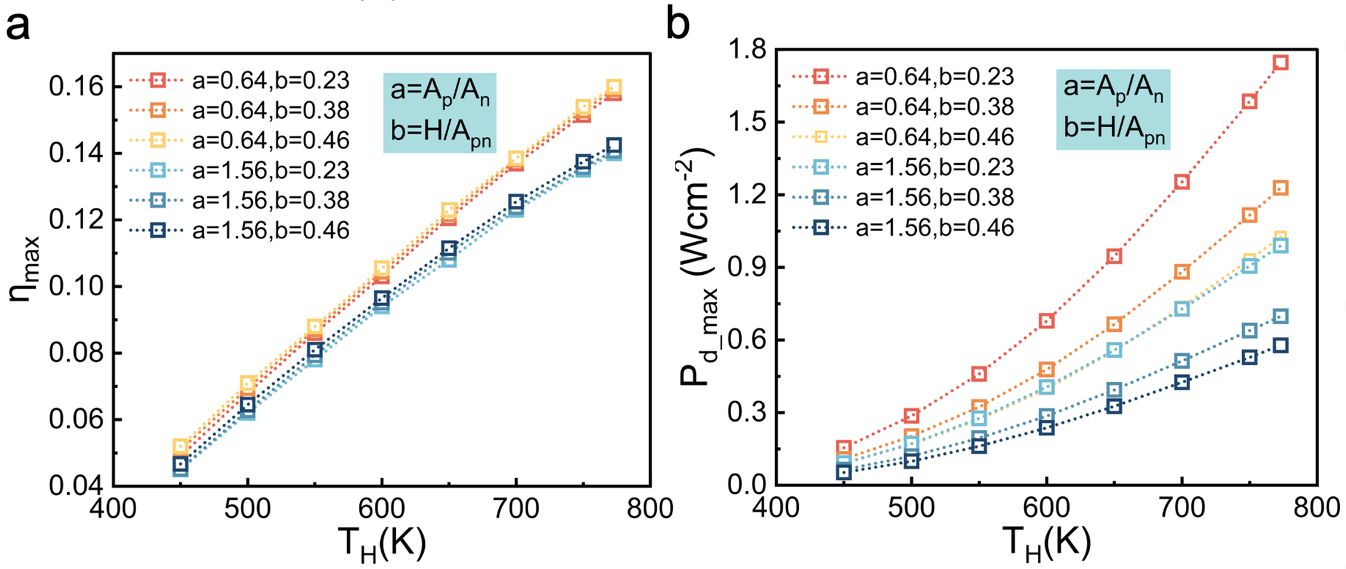


**Fig. S4.** (a) The maximum conversion efficiency $\eta$_max_ and (b) the maximum power density *P*_d_max_ of the segmented devices with different geometric parameters change with the hot side temperature *T*_H_.


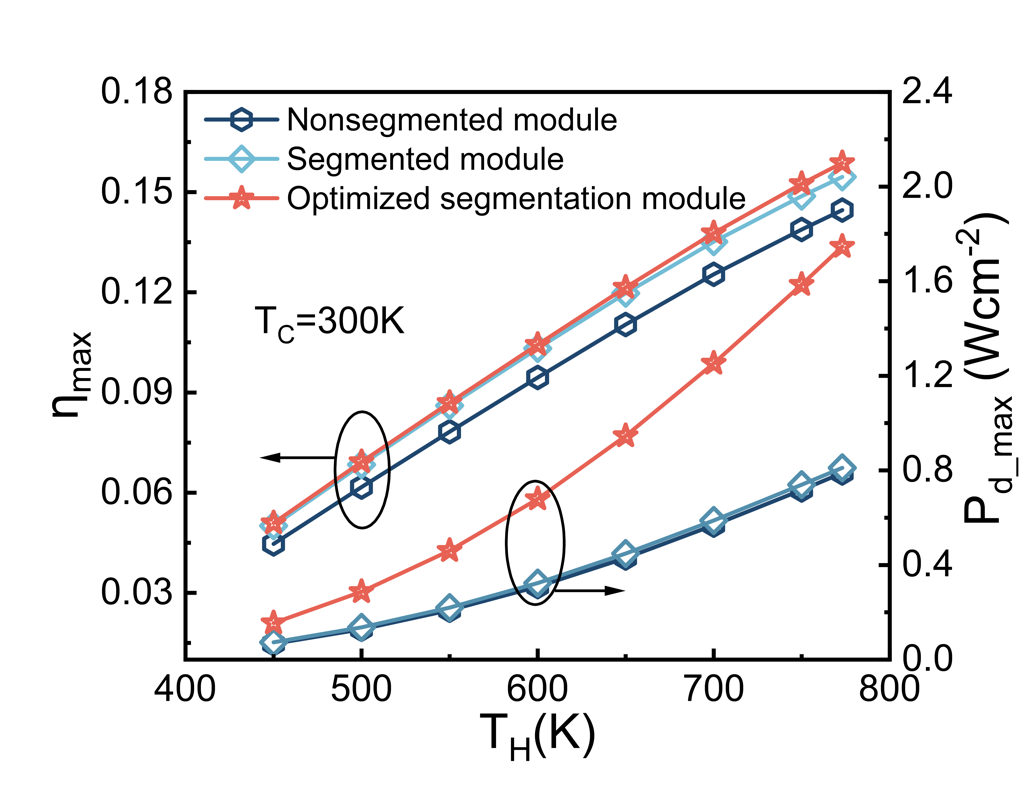


**Fig. S5.** The finite element simulation results of the maximum conversion efficiency $\eta$_max_ and the maximum power density *P*_d_max_ of the non-segmented power generation unit, the segmented power generation unit and the optimized segmented power generation unit vary with the hot side temperature *T*_H_.


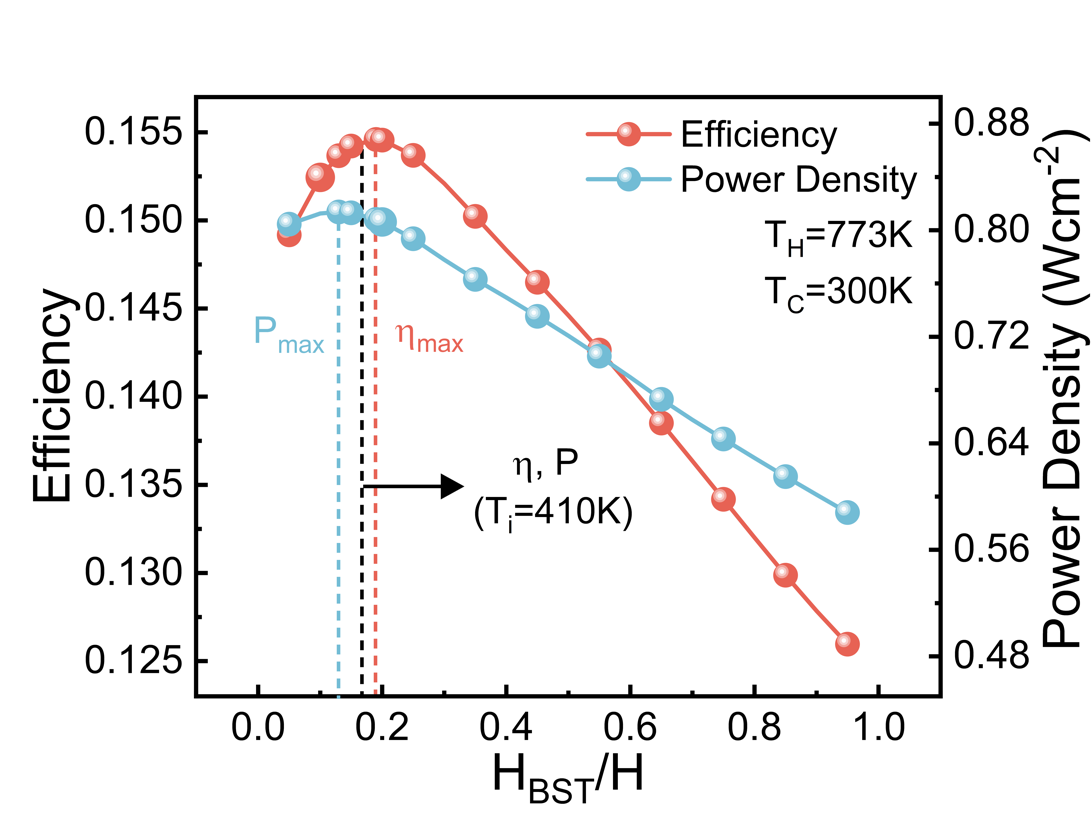


**Fig. S6.** Under the constant thermal boundary conditions (*T*_H_ = 773 K, *T*_C_ = 300 K), the maximum efficiency and maximum power density change with the segmentation ratio.


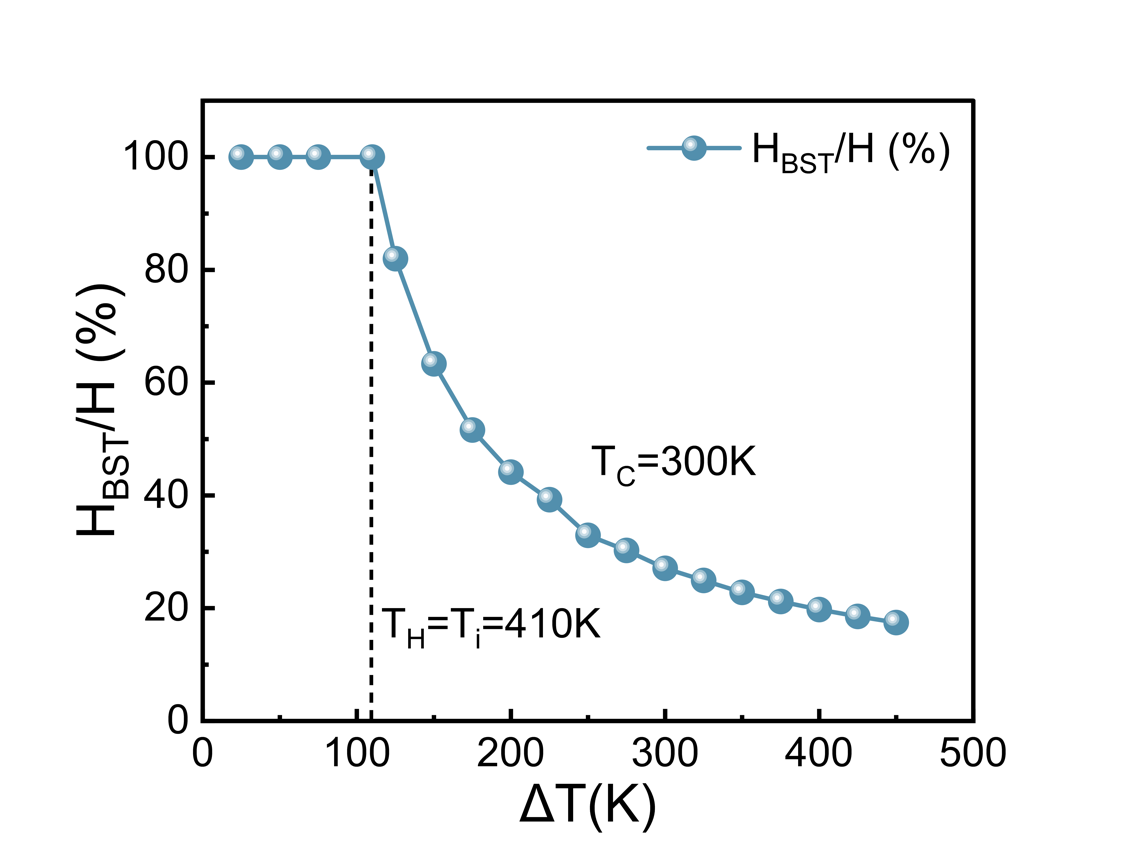


**Fig. S7.** The relationship between the height ratio of the cold side material in the whole section of the segmented power generation unit and the temperature difference between the cold and hot side.


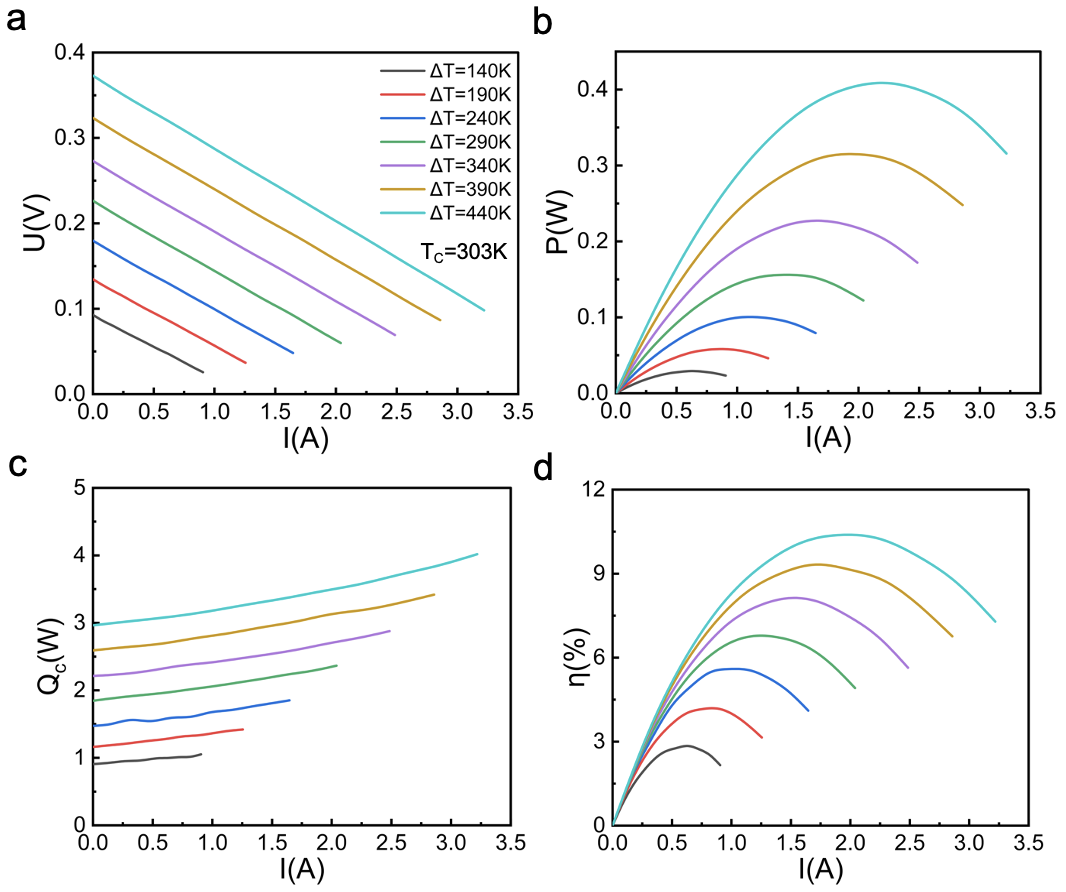


**Fig. S8.** The performance of 2-pair module is measured. (a) *U*-*I*, (b) *P*-*I*, (c) *Q*_c_-*I*, (d) $\eta$-*I*. The cold side temperature (*T*_C_) is fixed at 303 K, and the temperature difference ($\Delta T$) ranges from 140 K to 440 K.


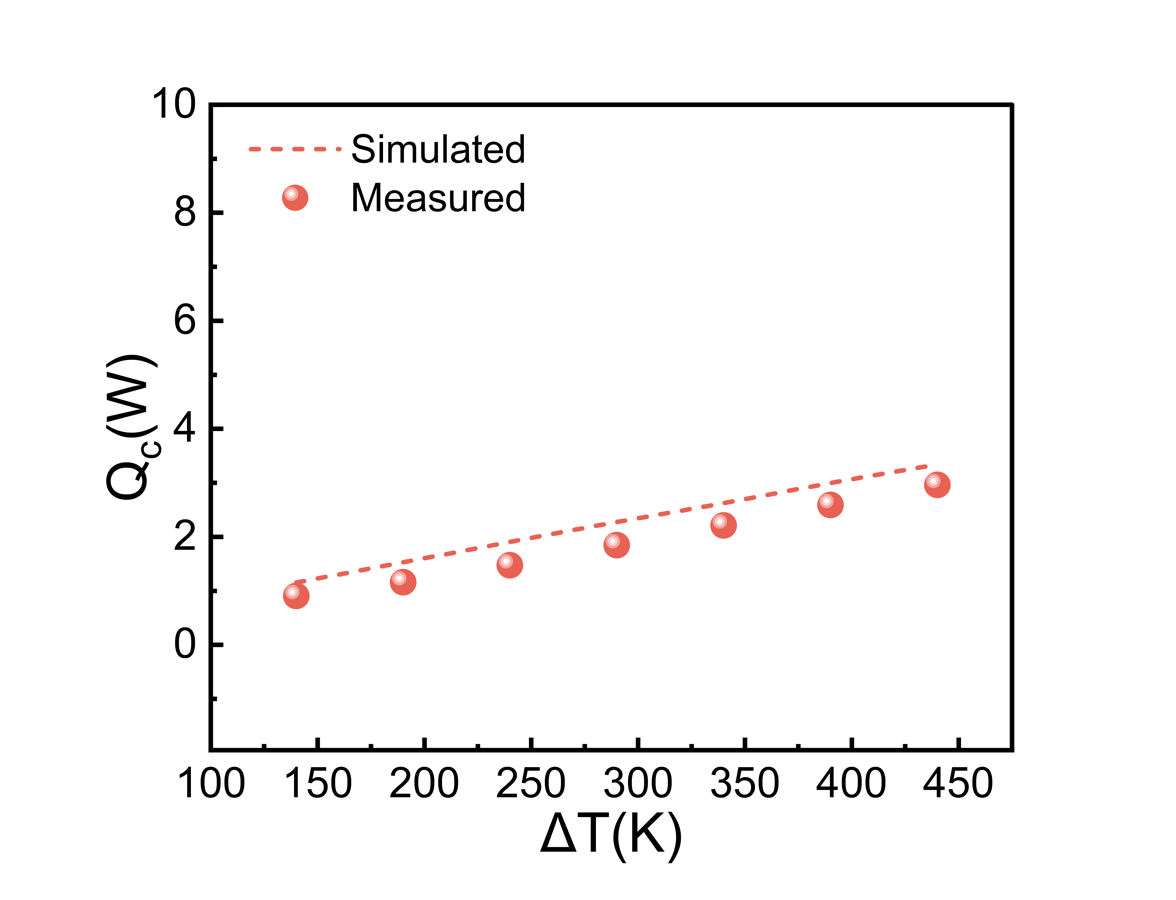


**Fig. S9.** Heat flow of the cold-side (*Q*_c_) under varying temperature differentials.

**
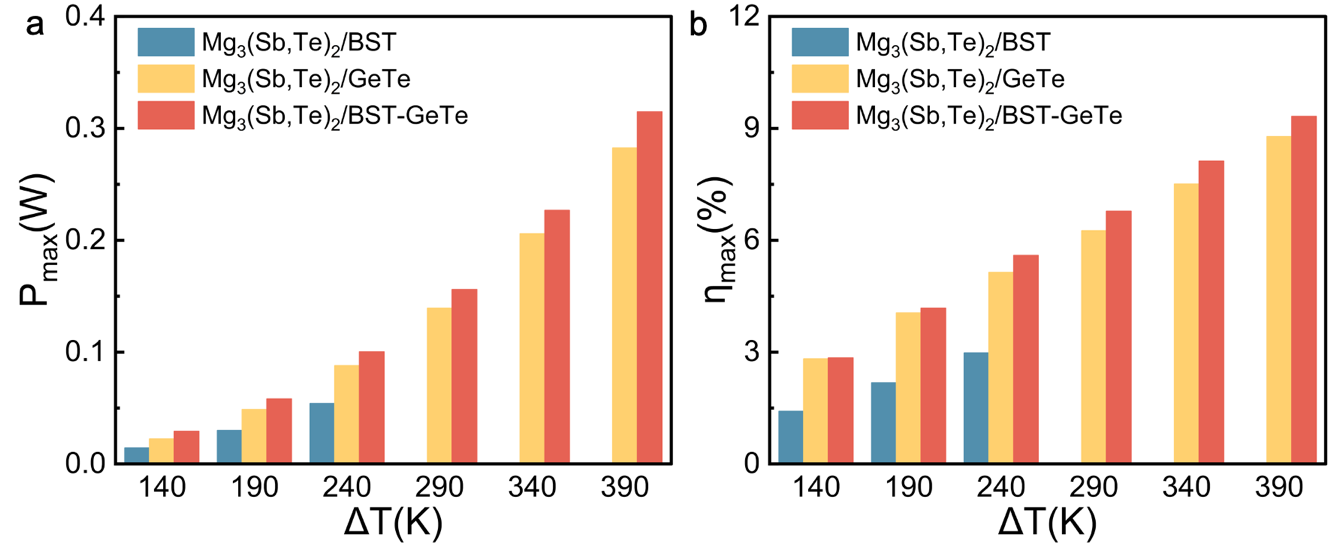
**

**Fig. S10.** Comparison of output power and conversion efficiency between segmented and unsegmented devices.

**
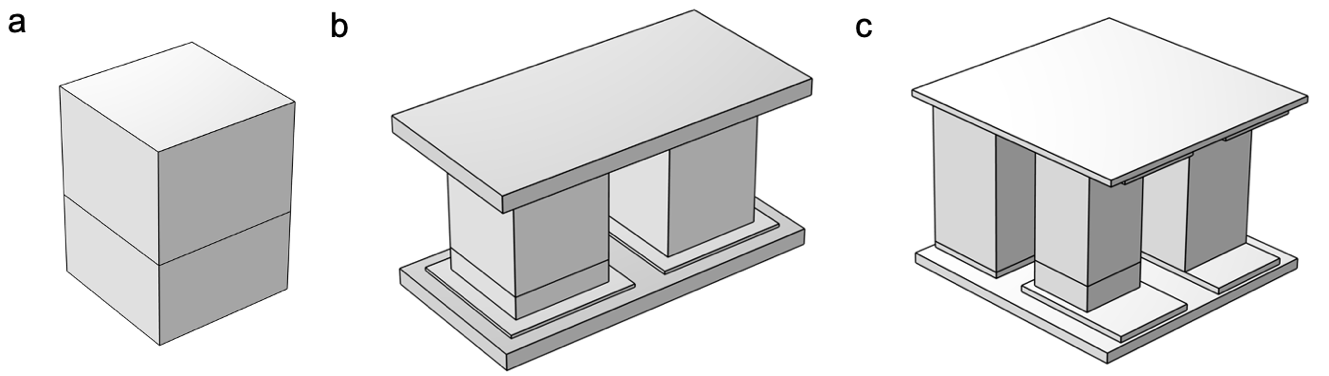
**

**Fig. S11.** Model for finite element simulation. (a) Thermoelectric single leg, (b) thermal power unit and (c) 2-pair module.


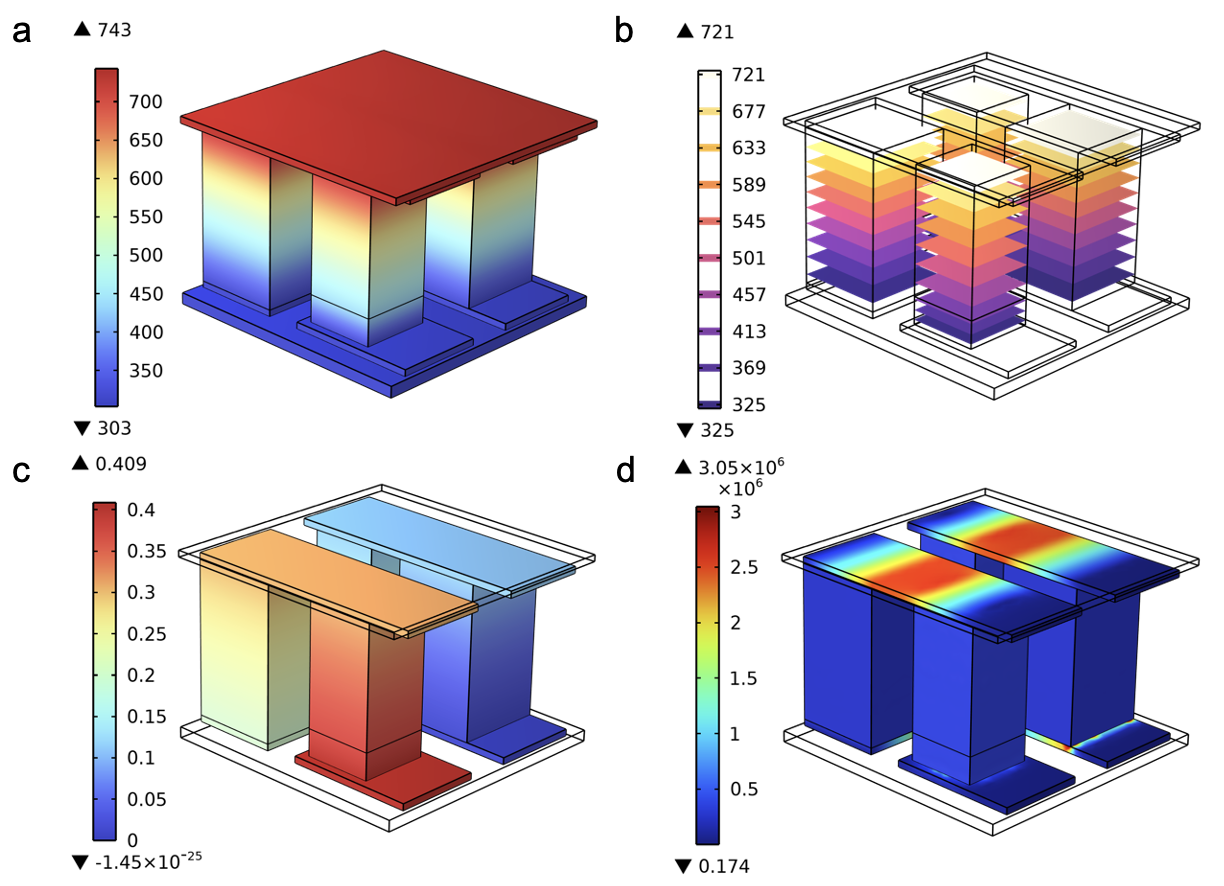


**Fig. S12.** Simulation results of 2-pair module. (a) Temperature distribution (K), (b) Temperature isosurface (K), (c) potential distribution (V) and (d) current density (A m^-2^).

**References**

1. Y. Sun, J. Fu, Y. Ohishi, K. Toh, K. Suekuni, K. Kihou, U. Anazawa, C.-H. Lee, K. Kurosaki, *ACS Appl. Mater. Interfaces* **2023**, *15*, 23246.
2. C. Xu, Z. Liang, W. Ren, S.Song, F. Zhang, Z. Ren, *Adv. Energy Mater.* **2022**, *12*, 2202392.
3. S. Wan, Q. Song, H. Chen, Q. Zhang, J. Liao, X. Xia, C. Wang, P. Qiu, B. Chen, S. Bai, L. Chen, *Cell Rep. Phys. Sci.* **2023**, *4*, 101651.
4. V. Ravi, S. Firdosy, T. Caillat, E. Brandon, K. Van Der Walde, L. Maricic, A. Sayir, *J. Electron. Mater.* **2009**, *38*, 1433.
5. W. Silpawilawan, K. Kurosaki, Y. Ohishi, H. Muta, S. Yamanaka, *J. Mater. Chem. C* **2017**, *5*, 6677.
